# (3,5-Dimethyl-1*H*-pyrazol-1-yl){3-[(3,5-dimethyl-1*H*-pyrazol-1-yl)carbon­yl]-5-methyl­indolizin-1-yl}methanone

**DOI:** 10.1107/S1600536813005060

**Published:** 2013-02-28

**Authors:** Xiang-Yun Song, Wei-Jin Gu, Yu-Liang Jiang, Bing-Xiang Wang

**Affiliations:** a Department of Applied Chemistry, Nanjing Normal University, Nanjing 210097, People’s Republic of China; b Key Laboratory of Applied Photochemistry, Nanjing Normal University, Nanjing 210097, People’s Republic of China; c Jiangsu Key Laboratory of Biofunctional Materials, Jiangsu Research Center of Biomedical Functional Materials Engineering, Nanjing Normal University, Nanjing 210097, People’s Republic of China

## Abstract

There are two independent mol­ecules in the asymmetric unit of the title compound, C_21_H_21_N_5_O_2_. In each mol­ecule, the indolizine ring system is essentially planar, with r.m.s. deviations of 0.030 and 0.028 Å. The dihedral angles between the indolizine ring system and the pyrazole rings are 54.7 (3) and 8.6 (3)° in one mol­ecule and 54.4 (3) and 6.6 (3)° in the other. In the crystal, weak C—H⋯O and C—H⋯N hydrogen bonds link mol­ecules, forming a two-dimensional network parallel to (100).

## Related literature

For the biological applications of indolizines and pyrazoles, see: Tukulula *et al.* (2010 ▶); James *et al.* (2008 ▶); Teklu *et al.* (2005 ▶); McDonald *et al.* (2006 ▶); Jagerovic *et al.* (2002 ▶). For background to and the synthesis of related hetrocycles, see: Gu *et al.* (2011 ▶); Shen *et al.* (2006 ▶, 2008 ▶); Wang, *et al.* (2000 ▶).
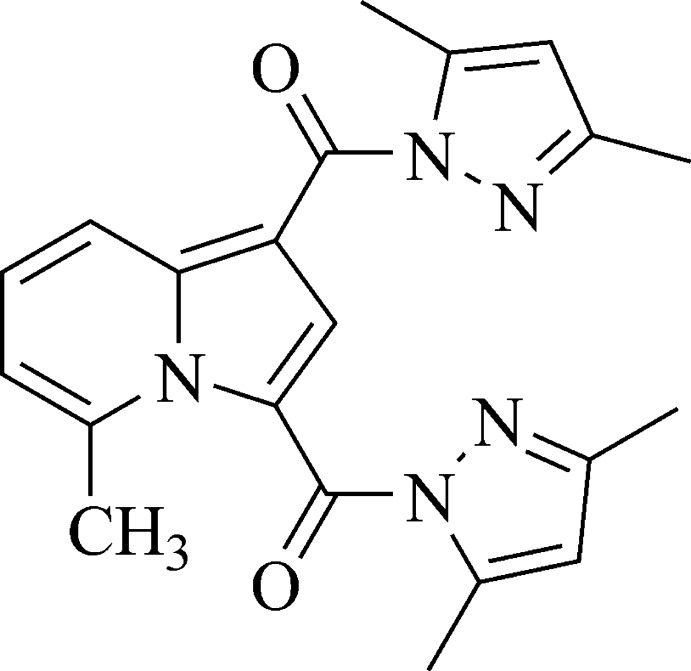


## Experimental

### 

#### Crystal data

C_21_H_21_N_5_O_2_
*M* *_r_* = 375.43
Orthorhombic, 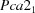

*a* = 19.7286 (11) Å
*b* = 11.5659 (14) Å
*c* = 17.8088 (18) Å
*V* = 4063.6 (7) Å^3^
*Z* = 8
Mo *K*α radiation
μ = 0.08 mm^−1^
*T* = 291 K
0.26 × 0.22 × 0.20 mm

#### Data collection

Bruker SMART APEX CCD diffractometer
Absorption correction: multi-scan (*SADABS*; Bruker, 2000 ▶) *T*_min_ = 0.979, *T*_max_ = 0.984
30693 measured reflections
7955 independent reflections
6221 reflections with *I* > 2σ(*I*)
*R*_int_ = 0.025

#### Refinement

*R*[*F* ^2^ > 2σ(*F* ^2^)] = 0.050
*wR*(*F* ^2^) = 0.129
*S* = 1.07
7955 reflections
516 parameters
1 restraint
H-atom parameters constrained
Δρ_max_ = 0.19 e Å^−3^
Δρ_min_ = −0.25 e Å^−3^

### 

Data collection: *SMART* (Bruker, 2000 ▶); cell refinement: *SAINT* (Bruker, 2000 ▶); data reduction: *SAINT*; program(s) used to solve structure: *SHELXTL* (Sheldrick, 2008 ▶); program(s) used to refine structure: *SHELXTL*; molecular graphics: *SHELXTL*; software used to prepare material for publication: *SHELXTL*.

## Supplementary Material

Crystal structure: contains datablock(s) global, I. DOI: 10.1107/S1600536813005060/lh5581sup1.cif

Supplementary material file. DOI: 10.1107/S1600536813005060/lh5581Isup3.cdx

Structure factors: contains datablock(s) I. DOI: 10.1107/S1600536813005060/lh5581Isup4.hkl

Supplementary material file. DOI: 10.1107/S1600536813005060/lh5581Isup5.cml

Additional supplementary materials:  crystallographic information; 3D view; checkCIF report

## Figures and Tables

**Table 1:** Hydrogen-bond geometry (Å, °)

*D*—H⋯*A*	*D*—H	H⋯*A*	*D*⋯*A*	*D*—H⋯*A*
C12—H12⋯O2^i^	0.93	2.37	3.282 (4)	167
C21—H21*B*⋯O1^ii^	0.96	2.59	3.404 (4)	142
C30—H30*C*⋯N3^iii^	0.96	2.52	3.472 (4)	169
C33—H33⋯O4^iv^	0.93	2.55	3.393 (4)	152

Symmetry codes: (i) 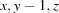; (ii) 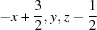; (iii) 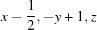; (iv) 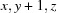.

**Fig. 1.**
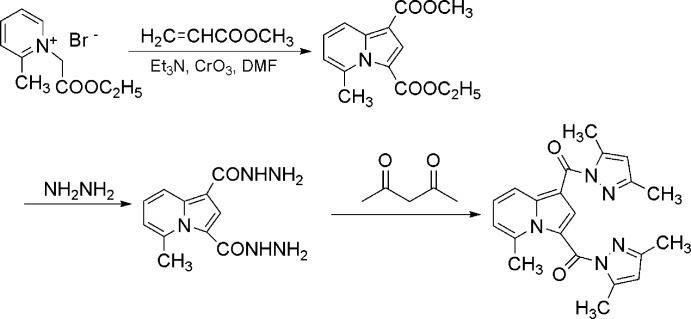
The reaction scheme.

**Fig. 2.**
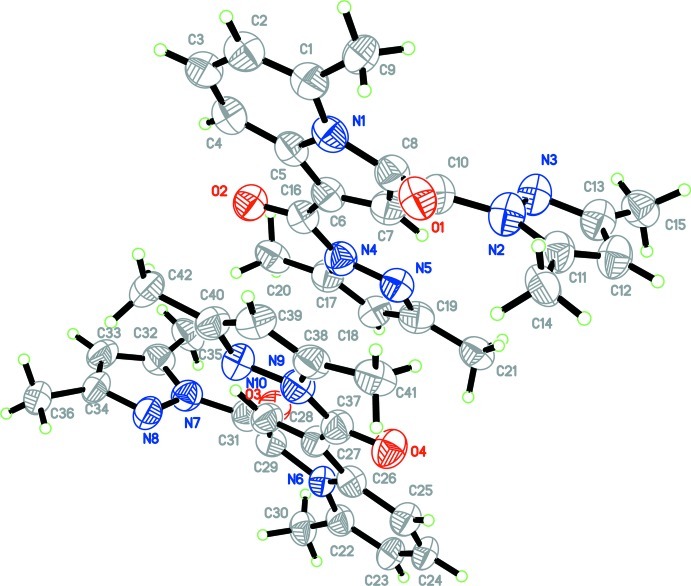
The asymmetric unit of title compound. Displacement ellipsoids at the 50% probability level.

## supplementary crystallographic information

### Comment

Indolizines and pyrazoles are important classes of bio-active drug targets in the pharmaceutical industry, as they are the core structure of numerous biologically active compounds (Tukulula *et al.*, 2010; James *et al.*, 2008; Teklu *et al.*, 2005; McDonald *et al.*, 2006; Jagerovic *et al.*, 2002). In our continuing studies on the synthesis and properties of heterocycles (Gu *et al.*, 2011; Shen *et al.*, 2008; Shen *et al.*, 2006; Wang, *et al.*, 2000) we have prepared (Fig. 1) and determined the crystal structure of the title compound .

The molecular structure of the title compound is shown in Fig. 2. There are two independent molecules is the asymmetric unit. The r.m.s deviation for the indolizine rings systems N1/C1-C8 and N6/C22-C29 are 0.030 and 0.028 Å, respectively. The dihedral angle between N1/C1-C8 and N2/N3/C11-C13 is 125.3 (3) °, N1/C1-C8 and N4/N5/C17-C19 is 8.6 (3)°, N6/C22-C29 and N7/N8/C32-C34 is 125.6 (3)°, and N6/C22-C29 and N9/N10/C38-C40 = 6.6 (3)°. In the crystal weak C—H···O and C—H···N hydrogen bonds link molecules to form a two-dimensional network parallel to (100).

### Experimental

3-ethyl 1-methyl 5-methylindolizine-1,3-dicarboxylate was prepared through 1,3-dipolar cycloaddition according to a procedure described in the literature (Wang, *et al.*, 2000). A suspension of N-(carbethoxymethyl)-2-ethylpyridinium bromide (C_6_H_7_N^+^CH_2_COOC_2_H_5_.Br^-^) (10 mmol), methyl acrylate (40 mmol), Et_3_N (60 ml) and CrO_3_ (80 mmol) in DMF (40 ml) was stirred at 363K for 4 h (monitored by TLC). The mixture was cooled to room temperature and poured into 5% aqueous HCl (150 mL). The deep brown power was collected by filtration and washed with ethanol (20 mL) After drying the solid was collected 1.32 g (51%).

3-ethyl 1-methyl 5-methylindolizine-1,3-dicarboxylate (5 mmol) was dissolved in 6 ml of ethanol and 30 ml 80% N_2_H_4_.H_2_O (45 mmol) was added dropwise. The solution was refluxed for 8 h and cooled to yield the product, 0.69 g (56%) as 5-methylindolizine-1,3-dicarbohydrazide.

5-methylindolizine-1,3-dicarbohydrazide (1 mmol) was dissolved in 2 ml acetic acid, then acetylacetone (4 mmol, dissolved in 4ml ethanol) was added. After stirring for 4 h, the mixture was purified by chromatography [silica gel, 20% ethyl acetate in petroleum ether (60 C90)] to yield colorless block crystals of the title compound, 0.26 g (70%). 1*H*-NMR (CDCl_3_, 400 MHz): 2.27 (s, 3H, –CH3), 2.31 (s, 3H, –CH3), 2.63 (s, 3H, –CH3), 2.64 (s, 6H, –CH3, –CH3), 6.02 (s, 1H, pyrazole =CH), 6.10(s, 1H, pyrazole =CH), 6.94 (d, 1H, indolizine =CH), 7.45 (t, 1H, indolizine =CH), 8.52 (s, 1H, indolizine =CH), 8.55 (d, 1H, indolizine =CH).

### Refinement

H atoms were placed in calculated positions with C—H = 0.93 and 0.96Å. They were included in a riding-motion approximation with U_iso_(H) = 1.2 U_eq_(C) or 1.5 U_eq_(C_methyl_).

### Crystal data

**Table:**

C_21_H_21_N_5_O_2_	*F*(000) = 1584
*M**_r_* = 375.43	*D*_x_ = 1.227 Mg m^−^^3^
Orthorhombic, *P**c**a*2_1_	Mo *K*α radiation, λ = 0.71073 Å
Hall symbol: P 2c -2ac	Cell parameters from 5647 reflections
*a* = 19.7286 (11) Å	θ = 2.3–21.7°
*b* = 11.5659 (14) Å	µ = 0.08 mm^−^^1^
*c* = 17.8088 (18) Å	*T* = 291 K
*V* = 4063.6 (7) Å^3^	Block, colourless
*Z* = 8	0.26 × 0.22 × 0.20 mm

### Data collection

**Table:**

Bruker SMART APEX CCD diffractometer	7955 independent reflections
Radiation source: sealed tube	6221 reflections with *I* > 2σ(*I*)
Graphite monochromator	*R*_int_ = 0.025
φ and ω scans	θ_max_ = 26.0°, θ_min_ = 1.8°
Absorption correction: multi-scan (*SADABS*; Bruker, 2000)	*h* = −24→23
*T*_min_ = 0.979, *T*_max_ = 0.984	*k* = −12→14
30693 measured reflections	*l* = −21→21

### Refinement

**Table:**

Refinement on *F*^2^	Primary atom site location: structure-invariant direct methods
Least-squares matrix: full	Secondary atom site location: difference Fourier map
*R*[*F*^2^ > 2σ(*F*^2^)] = 0.050	Hydrogen site location: inferred from neighbouring sites
*wR*(*F*^2^) = 0.129	H-atom parameters constrained
*S* = 1.07	*w* = 1/[σ^2^(*F*_o_^2^) + (0.05*P*)^2^ + 1.55*P*] where *P* = (*F*_o_^2^ + 2*F*_c_^2^)/3
7955 reflections	(Δ/σ)_max_ < 0.001
516 parameters	Δρ_max_ = 0.19 e Å^−^^3^
1 restraint	Δρ_min_ = −0.25 e Å^−^^3^

### Special details

**Table:**

Geometry. All esds (except the esd in the dihedral angle between two l.s. planes) are estimated using the full covariance matrix. The cell esds are taken into account individually in the estimation of esds in distances, angles and torsion angles; correlations between esds in cell parameters are only used when they are defined by crystal symmetry. An approximate (isotropic) treatment of cell esds is used for estimating esds involving l.s. planes.
Refinement. Refinement of F^2^ against ALL reflections. The weighted R-factor wR and goodness of fit S are based on F^2^, conventional R-factors R are based on F, with F set to zero for negative F^2^. The threshold expression of F^2^ > 2sigma(F^2^) is used only for calculating R-factors(gt) etc. and is not relevant to the choice of reflections for refinement. R-factors based on F^2^ are statistically about twice as large as those based on F, and R- factors based on ALL data will be even larger.

### Fractional atomic coordinates and isotropic or equivalent isotropic displacement parameters (Å^2^)

**Table:**

	*x*	*y*	*z*	*U*_iso_*/*U*_eq_	
C1	0.95788 (15)	0.4268 (3)	0.79194 (16)	0.0511 (7)	
C2	0.97669 (16)	0.5313 (3)	0.81916 (17)	0.0537 (8)	
H2	1.0113	0.5345	0.8545	0.064*	
C3	0.94637 (15)	0.6336 (3)	0.79647 (17)	0.0504 (7)	
H3	0.9599	0.7036	0.8174	0.061*	
C4	0.89665 (16)	0.6313 (3)	0.74333 (17)	0.0517 (7)	
H4	0.8786	0.7003	0.7254	0.062*	
C5	0.87266 (15)	0.5260 (3)	0.71560 (16)	0.0470 (7)	
C6	0.82339 (15)	0.4954 (3)	0.66095 (15)	0.0471 (7)	
C7	0.82505 (15)	0.3748 (3)	0.65706 (16)	0.0502 (7)	
H7	0.7984	0.3307	0.6249	0.060*	
C8	0.87156 (15)	0.3292 (3)	0.70735 (17)	0.0479 (7)	
C9	0.99612 (15)	0.3185 (2)	0.80885 (16)	0.0487 (7)	
H9A	0.9704	0.2717	0.8431	0.073*	
H9B	1.0389	0.3378	0.8312	0.073*	
H9C	1.0037	0.2763	0.7632	0.073*	
C10	0.86911 (16)	0.2129 (3)	0.73751 (17)	0.0506 (7)	
C11	0.83518 (16)	0.0113 (3)	0.70175 (17)	0.0524 (7)	
C12	0.82865 (17)	−0.0416 (3)	0.63537 (17)	0.0544 (8)	
H12	0.8144	−0.1173	0.6278	0.065*	
C13	0.84745 (16)	0.0390 (3)	0.57820 (18)	0.0542 (8)	
C14	0.82015 (17)	−0.0311 (3)	0.77875 (18)	0.0588 (8)	
H14A	0.7998	−0.1064	0.7758	0.088*	
H14B	0.7895	0.0213	0.8031	0.088*	
H14C	0.8615	−0.0358	0.8070	0.088*	
C15	0.85063 (17)	0.0238 (3)	0.49569 (17)	0.0569 (8)	
H15A	0.8706	0.0912	0.4733	0.085*	
H15B	0.8057	0.0132	0.4763	0.085*	
H15C	0.8777	−0.0428	0.4840	0.085*	
C16	0.78392 (15)	0.5808 (3)	0.62317 (16)	0.0483 (7)	
C17	0.69986 (16)	0.6164 (3)	0.51854 (18)	0.0532 (7)	
C18	0.66236 (15)	0.5437 (3)	0.47619 (16)	0.0489 (7)	
H18	0.6326	0.5646	0.4381	0.059*	
C19	0.67648 (15)	0.4313 (3)	0.50014 (18)	0.0533 (7)	
C20	0.70343 (15)	0.7452 (2)	0.51772 (18)	0.0523 (8)	
H20A	0.6759	0.7745	0.4776	0.078*	
H20B	0.7496	0.7690	0.5104	0.078*	
H20C	0.6871	0.7749	0.5647	0.078*	
C21	0.64842 (16)	0.3191 (3)	0.47238 (17)	0.0546 (7)	
H21A	0.6708	0.2563	0.4974	0.082*	
H21B	0.6558	0.3131	0.4192	0.082*	
H21C	0.6007	0.3159	0.4827	0.082*	
C22	0.41826 (16)	0.5455 (3)	0.49679 (16)	0.0493 (7)	
C23	0.39368 (16)	0.4389 (3)	0.48031 (17)	0.0527 (8)	
H23	0.3575	0.4325	0.4471	0.063*	
C24	0.42111 (17)	0.3377 (3)	0.51186 (18)	0.0545 (8)	
H24	0.4041	0.2658	0.4980	0.065*	
C25	0.47187 (16)	0.3449 (3)	0.56205 (18)	0.0537 (7)	
H25	0.4883	0.2783	0.5849	0.064*	
C26	0.50012 (16)	0.4536 (3)	0.57998 (17)	0.0511 (7)	
C27	0.55051 (15)	0.4910 (3)	0.63101 (16)	0.0474 (7)	
C28	0.55515 (15)	0.6100 (3)	0.62361 (16)	0.0478 (7)	
H28	0.5846	0.6570	0.6507	0.057*	
C29	0.51025 (14)	0.6481 (3)	0.57084 (17)	0.0480 (7)	
C30	0.38317 (16)	0.6508 (3)	0.47244 (16)	0.0529 (7)	
H30A	0.3402	0.6306	0.4509	0.079*	
H30B	0.4102	0.6902	0.4356	0.079*	
H30C	0.3762	0.7006	0.5149	0.079*	
C31	0.51566 (15)	0.7556 (3)	0.52786 (17)	0.0503 (7)	
C32	0.55300 (16)	0.9622 (3)	0.54312 (16)	0.0525 (7)	
C33	0.55914 (16)	1.0296 (3)	0.60368 (18)	0.0557 (8)	
H33	0.5725	1.1067	0.6042	0.067*	
C34	0.54178 (16)	0.9629 (3)	0.66563 (18)	0.0544 (8)	
C35	0.56546 (17)	0.9886 (3)	0.46158 (17)	0.0570 (8)	
H35A	0.5848	1.0644	0.4570	0.086*	
H35B	0.5233	0.9856	0.4347	0.086*	
H35C	0.5962	0.9325	0.4411	0.086*	
C36	0.53860 (17)	0.9990 (3)	0.74706 (17)	0.0555 (8)	
H36A	0.5834	1.0164	0.7647	0.083*	
H36B	0.5199	0.9372	0.7764	0.083*	
H36C	0.5105	1.0663	0.7518	0.083*	
C37	0.58944 (16)	0.4080 (3)	0.67483 (17)	0.0502 (7)	
C38	0.68154 (15)	0.3805 (3)	0.77224 (18)	0.0501 (7)	
C39	0.71764 (15)	0.4592 (3)	0.81277 (17)	0.0488 (7)	
H39	0.7507	0.4425	0.8484	0.059*	
C40	0.69609 (16)	0.5699 (3)	0.79131 (17)	0.0504 (7)	
C41	0.68581 (16)	0.2528 (2)	0.77334 (18)	0.0551 (8)	
H41A	0.6442	0.2214	0.7925	0.083*	
H41B	0.7227	0.2293	0.8050	0.083*	
H41C	0.6932	0.2247	0.7233	0.083*	
C42	0.71876 (17)	0.6851 (3)	0.81811 (18)	0.0525 (7)	
H42A	0.6845	0.7179	0.8499	0.079*	
H42B	0.7262	0.7350	0.7758	0.079*	
H42C	0.7602	0.6769	0.8459	0.079*	
N1	0.90253 (12)	0.4237 (2)	0.74381 (13)	0.0477 (6)	
N2	0.85490 (14)	0.1237 (2)	0.68550 (15)	0.0589 (7)	
N3	0.86355 (13)	0.1389 (2)	0.60984 (14)	0.0518 (6)	
N4	0.73572 (13)	0.5457 (2)	0.56655 (14)	0.0507 (6)	
N5	0.72100 (12)	0.4309 (2)	0.55560 (13)	0.0478 (6)	
N6	0.47486 (12)	0.5511 (2)	0.54330 (13)	0.0465 (6)	
N7	0.53127 (12)	0.8556 (2)	0.56983 (14)	0.0497 (6)	
N8	0.52484 (13)	0.8577 (2)	0.64599 (14)	0.0520 (6)	
N9	0.63732 (13)	0.4465 (2)	0.72789 (14)	0.0481 (6)	
N10	0.64724 (13)	0.5633 (2)	0.74015 (14)	0.0515 (6)	
O1	0.87568 (11)	0.18868 (18)	0.80311 (12)	0.0559 (5)	
O2	0.78751 (10)	0.68345 (17)	0.63626 (12)	0.0531 (5)	
O3	0.51202 (11)	0.76188 (18)	0.46034 (12)	0.0556 (5)	
O4	0.58222 (10)	0.30270 (18)	0.66841 (12)	0.0559 (5)	

### Atomic displacement parameters (Å^2^)

**Table:**

	*U*^11^	*U*^22^	*U*^33^	*U*^12^	*U*^13^	*U*^23^
C1	0.0487 (16)	0.0568 (19)	0.0479 (17)	0.0042 (14)	0.0005 (13)	0.0006 (14)
C2	0.0537 (18)	0.060 (2)	0.0475 (16)	0.0095 (16)	−0.0077 (14)	0.0062 (15)
C3	0.0493 (17)	0.0514 (18)	0.0507 (17)	0.0049 (13)	−0.0012 (14)	0.0048 (14)
C4	0.0563 (17)	0.0508 (18)	0.0479 (16)	0.0081 (14)	0.0041 (14)	0.0021 (14)
C5	0.0477 (16)	0.0447 (17)	0.0487 (16)	0.0059 (13)	0.0069 (13)	0.0018 (13)
C6	0.0491 (16)	0.0463 (16)	0.0458 (16)	0.0060 (13)	0.0026 (13)	0.0044 (13)
C7	0.0487 (17)	0.0537 (18)	0.0483 (17)	0.0001 (14)	0.0011 (14)	0.0025 (14)
C8	0.0518 (17)	0.0440 (17)	0.0481 (16)	0.0029 (13)	0.0025 (13)	0.0031 (13)
C9	0.0539 (17)	0.0483 (17)	0.0437 (15)	−0.0058 (13)	−0.0063 (14)	−0.0008 (13)
C10	0.0561 (17)	0.0475 (17)	0.0483 (17)	0.0033 (13)	−0.0022 (14)	0.0007 (14)
C11	0.0575 (18)	0.0504 (18)	0.0494 (17)	0.0066 (14)	−0.0094 (14)	−0.0073 (14)
C12	0.067 (2)	0.0446 (17)	0.0517 (17)	0.0039 (14)	−0.0111 (15)	−0.0017 (14)
C13	0.0612 (19)	0.0468 (17)	0.0547 (18)	0.0058 (15)	−0.0136 (15)	0.0012 (14)
C14	0.0580 (18)	0.0523 (18)	0.066 (2)	0.0113 (15)	0.0160 (16)	−0.0099 (16)
C15	0.0592 (18)	0.0564 (19)	0.0552 (18)	0.0045 (15)	0.0096 (15)	0.0069 (15)
C16	0.0484 (17)	0.0477 (18)	0.0488 (17)	0.0028 (13)	0.0020 (13)	−0.0016 (14)
C17	0.0559 (18)	0.0517 (18)	0.0520 (17)	−0.0022 (15)	0.0046 (15)	−0.0007 (14)
C18	0.0502 (17)	0.0511 (18)	0.0453 (16)	−0.0023 (13)	−0.0048 (13)	0.0042 (13)
C19	0.0468 (16)	0.058 (2)	0.0550 (18)	0.0021 (14)	−0.0013 (15)	0.0055 (15)
C20	0.0523 (17)	0.0487 (17)	0.0559 (18)	0.0135 (14)	0.0115 (14)	0.0164 (14)
C21	0.0525 (17)	0.0573 (19)	0.0539 (17)	0.0059 (15)	0.0098 (14)	0.0044 (15)
C22	0.0558 (17)	0.0493 (18)	0.0427 (15)	−0.0023 (14)	0.0064 (14)	−0.0066 (13)
C23	0.0522 (17)	0.0539 (19)	0.0519 (18)	−0.0085 (15)	−0.0076 (14)	−0.0168 (15)
C24	0.0609 (18)	0.0454 (18)	0.0572 (18)	−0.0133 (14)	0.0017 (15)	−0.0182 (14)
C25	0.0562 (18)	0.0499 (17)	0.0552 (18)	−0.0071 (14)	0.0003 (15)	−0.0079 (14)
C26	0.0520 (17)	0.0531 (19)	0.0482 (16)	−0.0041 (14)	0.0097 (14)	−0.0098 (14)
C27	0.0509 (17)	0.0446 (16)	0.0468 (16)	−0.0083 (13)	0.0058 (13)	−0.0074 (13)
C28	0.0493 (16)	0.0452 (17)	0.0489 (16)	−0.0076 (13)	0.0021 (13)	−0.0002 (13)
C29	0.0463 (16)	0.0491 (17)	0.0486 (16)	−0.0036 (13)	0.0083 (13)	−0.0023 (13)
C30	0.0591 (18)	0.0541 (18)	0.0454 (16)	0.0058 (15)	−0.0070 (14)	−0.0125 (14)
C31	0.0545 (17)	0.0499 (18)	0.0466 (18)	0.0005 (14)	0.0022 (14)	−0.0026 (13)
C32	0.0569 (18)	0.0515 (19)	0.0492 (17)	−0.0020 (14)	0.0061 (14)	0.0090 (14)
C33	0.0571 (18)	0.0487 (17)	0.0613 (19)	−0.0173 (14)	0.0033 (16)	−0.0021 (15)
C34	0.0593 (19)	0.0457 (18)	0.0582 (18)	−0.0158 (14)	0.0006 (15)	−0.0120 (14)
C35	0.066 (2)	0.0476 (18)	0.0575 (18)	0.0112 (15)	0.0214 (16)	0.0127 (15)
C36	0.0614 (19)	0.0504 (18)	0.0549 (17)	−0.0115 (15)	0.0045 (15)	−0.0142 (14)
C37	0.0562 (18)	0.0437 (18)	0.0507 (17)	−0.0041 (14)	0.0051 (14)	−0.0004 (14)
C38	0.0477 (16)	0.0441 (17)	0.0584 (17)	0.0027 (14)	0.0088 (14)	0.0083 (14)
C39	0.0497 (16)	0.0548 (18)	0.0419 (15)	−0.0073 (14)	−0.0035 (13)	0.0146 (14)
C40	0.0499 (16)	0.0487 (18)	0.0525 (18)	−0.0090 (13)	0.0038 (14)	−0.0005 (14)
C41	0.0500 (16)	0.0517 (18)	0.0637 (19)	0.0089 (14)	0.0074 (14)	0.0190 (16)
C42	0.0588 (18)	0.0482 (17)	0.0506 (17)	−0.0168 (14)	−0.0049 (14)	−0.0025 (14)
N1	0.0485 (14)	0.0555 (15)	0.0391 (12)	0.0067 (11)	0.0028 (11)	0.0020 (11)
N2	0.0690 (18)	0.0496 (16)	0.0581 (17)	0.0013 (13)	−0.0064 (13)	−0.0029 (13)
N3	0.0610 (16)	0.0441 (14)	0.0503 (14)	0.0074 (11)	−0.0173 (12)	−0.0014 (11)
N4	0.0544 (15)	0.0466 (15)	0.0512 (14)	0.0010 (11)	0.0023 (12)	0.0004 (11)
N5	0.0492 (14)	0.0463 (14)	0.0479 (14)	0.0052 (11)	−0.0036 (11)	0.0062 (11)
N6	0.0497 (14)	0.0427 (14)	0.0472 (14)	−0.0042 (11)	0.0043 (11)	−0.0100 (11)
N7	0.0535 (14)	0.0441 (14)	0.0517 (14)	−0.0040 (11)	0.0011 (12)	0.0037 (11)
N8	0.0593 (15)	0.0440 (14)	0.0527 (15)	−0.0081 (12)	−0.0017 (12)	−0.0063 (11)
N9	0.0506 (13)	0.0431 (14)	0.0505 (14)	0.0012 (11)	0.0022 (11)	0.0038 (11)
N10	0.0580 (16)	0.0457 (15)	0.0506 (14)	−0.0040 (12)	−0.0046 (12)	−0.0042 (11)
O1	0.0595 (13)	0.0548 (12)	0.0535 (13)	0.0116 (10)	0.0011 (10)	−0.0043 (10)
O2	0.0549 (12)	0.0458 (13)	0.0587 (12)	0.0054 (9)	−0.0090 (10)	0.0019 (10)
O3	0.0619 (13)	0.0538 (13)	0.0512 (13)	−0.0047 (10)	0.0072 (10)	0.0049 (10)
O4	0.0599 (13)	0.0477 (13)	0.0602 (13)	0.0003 (10)	−0.0137 (11)	−0.0006 (10)

### Geometric parameters (Å, º)

**Table:**

C1—C2	1.354 (4)	C22—C30	1.467 (4)
C1—N1	1.389 (4)	C23—C24	1.406 (4)
C1—C9	1.493 (4)	C23—H23	0.9300
C2—C3	1.387 (4)	C24—C25	1.345 (4)
C2—H2	0.9300	C24—H24	0.9300
C3—C4	1.363 (4)	C25—C26	1.412 (4)
C3—H3	0.9300	C25—H25	0.9300
C4—C5	1.397 (4)	C26—N6	1.395 (4)
C4—H4	0.9300	C26—C27	1.415 (4)
C5—N1	1.414 (4)	C27—C28	1.386 (4)
C5—C6	1.420 (4)	C27—C37	1.456 (4)
C6—C7	1.397 (4)	C28—C29	1.365 (4)
C6—C16	1.427 (4)	C28—H28	0.9300
C7—C8	1.387 (4)	C29—N6	1.410 (4)
C7—H7	0.9300	C29—C31	1.463 (4)
C8—N1	1.411 (4)	C30—H30A	0.9600
C8—C10	1.448 (4)	C30—H30B	0.9600
C9—H9A	0.9600	C30—H30C	0.9600
C9—H9B	0.9600	C31—O3	1.207 (3)
C9—H9C	0.9600	C31—N7	1.411 (4)
C10—O1	1.209 (3)	C32—C33	1.337 (4)
C10—N2	1.415 (4)	C32—N7	1.389 (4)
C11—C12	1.337 (4)	C32—C35	1.504 (4)
C11—N2	1.387 (4)	C33—C34	1.389 (4)
C11—C14	1.486 (4)	C33—H33	0.9300
C12—C13	1.429 (4)	C34—N8	1.310 (4)
C12—H12	0.9300	C34—C36	1.510 (4)
C13—N3	1.324 (4)	C35—H35A	0.9600
C13—C15	1.481 (4)	C35—H35B	0.9600
C14—H14A	0.9600	C35—H35C	0.9600
C14—H14B	0.9600	C36—H36A	0.9600
C14—H14C	0.9600	C36—H36B	0.9600
C15—H15A	0.9600	C36—H36C	0.9600
C15—H15B	0.9600	C37—O4	1.231 (3)
C15—H15C	0.9600	C37—N9	1.408 (4)
C16—O2	1.212 (3)	C38—C39	1.363 (4)
C16—N4	1.444 (4)	C38—N9	1.403 (4)
C17—C18	1.350 (4)	C38—C41	1.480 (4)
C17—N4	1.379 (4)	C39—C40	1.402 (4)
C17—C20	1.491 (4)	C39—H39	0.9300
C18—C19	1.395 (4)	C40—N10	1.328 (4)
C18—H18	0.9300	C40—C42	1.484 (4)
C19—N5	1.322 (4)	C41—H41A	0.9600
C19—C21	1.495 (4)	C41—H41B	0.9600
C20—H20A	0.9600	C41—H41C	0.9600
C20—H20B	0.9600	C42—H42A	0.9600
C20—H20C	0.9600	C42—H42B	0.9600
C21—H21A	0.9600	C42—H42C	0.9600
C21—H21B	0.9600	N2—N3	1.370 (4)
C21—H21C	0.9600	N4—N5	1.373 (3)
C22—C23	1.357 (4)	N7—N8	1.362 (3)
C22—N6	1.392 (4)	N9—N10	1.382 (3)
			
C2—C1—N1	117.3 (3)	C26—C25—H25	120.0
C2—C1—C9	122.6 (3)	N6—C26—C25	118.2 (3)
N1—C1—C9	120.0 (3)	N6—C26—C27	107.7 (3)
C1—C2—C3	122.6 (3)	C25—C26—C27	134.0 (3)
C1—C2—H2	118.7	C28—C27—C26	106.8 (3)
C3—C2—H2	118.7	C28—C27—C37	132.2 (3)
C4—C3—C2	119.7 (3)	C26—C27—C37	120.9 (3)
C4—C3—H3	120.1	C29—C28—C27	110.1 (3)
C2—C3—H3	120.1	C29—C28—H28	125.0
C3—C4—C5	120.4 (3)	C27—C28—H28	125.0
C3—C4—H4	119.8	C28—C29—N6	107.7 (3)
C5—C4—H4	119.8	C28—C29—C31	126.0 (3)
C4—C5—N1	117.6 (3)	N6—C29—C31	122.0 (3)
C4—C5—C6	133.7 (3)	C22—C30—H30A	109.5
N1—C5—C6	108.7 (3)	C22—C30—H30B	109.5
C7—C6—C5	105.5 (2)	H30A—C30—H30B	109.5
C7—C6—C16	132.9 (3)	C22—C30—H30C	109.5
C5—C6—C16	121.6 (3)	H30A—C30—H30C	109.5
C8—C7—C6	111.3 (3)	H30B—C30—H30C	109.5
C8—C7—H7	124.3	O3—C31—N7	119.4 (3)
C6—C7—H7	124.3	O3—C31—C29	124.6 (3)
C7—C8—N1	106.8 (3)	N7—C31—C29	115.8 (3)
C7—C8—C10	124.8 (3)	C33—C32—N7	105.6 (3)
N1—C8—C10	124.3 (3)	C33—C32—C35	130.2 (3)
C1—C9—H9A	109.5	N7—C32—C35	124.2 (3)
C1—C9—H9B	109.5	C32—C33—C34	107.1 (3)
H9A—C9—H9B	109.5	C32—C33—H33	126.5
C1—C9—H9C	109.5	C34—C33—H33	126.5
H9A—C9—H9C	109.5	N8—C34—C33	111.5 (3)
H9B—C9—H9C	109.5	N8—C34—C36	120.2 (3)
O1—C10—N2	119.0 (3)	C33—C34—C36	128.2 (3)
O1—C10—C8	124.8 (3)	C32—C35—H35A	109.5
N2—C10—C8	116.2 (3)	C32—C35—H35B	109.5
C12—C11—N2	105.7 (3)	H35A—C35—H35B	109.5
C12—C11—C14	130.2 (3)	C32—C35—H35C	109.5
N2—C11—C14	123.9 (3)	H35A—C35—H35C	109.5
C11—C12—C13	107.8 (3)	H35B—C35—H35C	109.5
C11—C12—H12	126.1	C34—C36—H36A	109.5
C13—C12—H12	126.1	C34—C36—H36B	109.5
N3—C13—C12	109.2 (3)	H36A—C36—H36B	109.5
N3—C13—C15	121.0 (3)	C34—C36—H36C	109.5
C12—C13—C15	129.8 (3)	H36A—C36—H36C	109.5
C11—C14—H14A	109.5	H36B—C36—H36C	109.5
C11—C14—H14B	109.5	O4—C37—N9	116.9 (3)
H14A—C14—H14B	109.5	O4—C37—C27	122.8 (3)
C11—C14—H14C	109.5	N9—C37—C27	120.3 (2)
H14A—C14—H14C	109.5	C39—C38—N9	105.1 (3)
H14B—C14—H14C	109.5	C39—C38—C41	129.1 (3)
C13—C15—H15A	109.5	N9—C38—C41	125.9 (3)
C13—C15—H15B	109.5	C38—C39—C40	107.9 (3)
H15A—C15—H15B	109.5	C38—C39—H39	126.1
C13—C15—H15C	109.5	C40—C39—H39	126.1
H15A—C15—H15C	109.5	N10—C40—C39	110.7 (3)
H15B—C15—H15C	109.5	N10—C40—C42	119.4 (3)
O2—C16—C6	123.8 (3)	C39—C40—C42	129.8 (3)
O2—C16—N4	116.6 (3)	C38—C41—H41A	109.5
C6—C16—N4	119.6 (3)	C38—C41—H41B	109.5
C18—C17—N4	104.9 (3)	H41A—C41—H41B	109.5
C18—C17—C20	130.0 (3)	C38—C41—H41C	109.5
N4—C17—C20	125.1 (3)	H41A—C41—H41C	109.5
C17—C18—C19	107.4 (3)	H41B—C41—H41C	109.5
C17—C18—H18	126.3	C40—C42—H42A	109.5
C19—C18—H18	126.3	C40—C42—H42B	109.5
N5—C19—C18	111.4 (3)	H42A—C42—H42B	109.5
N5—C19—C21	119.3 (3)	C40—C42—H42C	109.5
C18—C19—C21	129.3 (3)	H42A—C42—H42C	109.5
C17—C20—H20A	109.5	H42B—C42—H42C	109.5
C17—C20—H20B	109.5	C1—N1—C8	130.1 (3)
H20A—C20—H20B	109.5	C1—N1—C5	121.7 (3)
C17—C20—H20C	109.5	C8—N1—C5	107.7 (2)
H20A—C20—H20C	109.5	N3—N2—C11	111.1 (3)
H20B—C20—H20C	109.5	N3—N2—C10	121.7 (3)
C19—C21—H21A	109.5	C11—N2—C10	127.1 (3)
C19—C21—H21B	109.5	C13—N3—N2	106.1 (3)
H21A—C21—H21B	109.5	N5—N4—C17	112.2 (2)
C19—C21—H21C	109.5	N5—N4—C16	120.7 (2)
H21A—C21—H21C	109.5	C17—N4—C16	127.1 (3)
H21B—C21—H21C	109.5	C19—N5—N4	104.1 (2)
C23—C22—N6	117.2 (3)	C22—N6—C26	121.9 (2)
C23—C22—C30	121.5 (3)	C22—N6—C29	129.9 (2)
N6—C22—C30	121.1 (3)	C26—N6—C29	107.7 (2)
C22—C23—C24	122.1 (3)	N8—N7—C32	110.7 (2)
C22—C23—H23	118.9	N8—N7—C31	121.4 (2)
C24—C23—H23	118.9	C32—N7—C31	127.8 (3)
C25—C24—C23	120.1 (3)	C34—N8—N7	105.0 (2)
C25—C24—H24	120.0	N10—N9—C38	110.8 (2)
C23—C24—H24	120.0	N10—N9—C37	120.7 (2)
C24—C25—C26	119.9 (3)	C38—N9—C37	128.5 (2)
C24—C25—H25	120.0	C40—N10—N9	105.5 (2)
			
N1—C1—C2—C3	−5.0 (5)	C7—C8—N1—C5	1.3 (3)
C9—C1—C2—C3	172.0 (3)	C10—C8—N1—C5	−156.7 (3)
C1—C2—C3—C4	−1.7 (5)	C4—C5—N1—C1	−5.9 (4)
C2—C3—C4—C5	4.7 (4)	C6—C5—N1—C1	172.6 (2)
C3—C4—C5—N1	−1.0 (4)	C4—C5—N1—C8	−179.0 (3)
C3—C4—C5—C6	−179.0 (3)	C6—C5—N1—C8	−0.5 (3)
C4—C5—C6—C7	177.7 (3)	C12—C11—N2—N3	−2.8 (4)
N1—C5—C6—C7	−0.4 (3)	C14—C11—N2—N3	−178.4 (3)
C4—C5—C6—C16	−2.8 (5)	C12—C11—N2—C10	−179.1 (3)
N1—C5—C6—C16	179.1 (3)	C14—C11—N2—C10	5.3 (5)
C5—C6—C7—C8	1.3 (3)	O1—C10—N2—N3	−163.3 (3)
C16—C6—C7—C8	−178.2 (3)	C8—C10—N2—N3	19.4 (4)
C6—C7—C8—N1	−1.6 (3)	O1—C10—N2—C11	12.6 (5)
C6—C7—C8—C10	156.2 (3)	C8—C10—N2—C11	−164.6 (3)
C7—C8—C10—O1	−132.8 (3)	C12—C13—N3—N2	−0.4 (3)
N1—C8—C10—O1	21.3 (5)	C15—C13—N3—N2	179.7 (3)
C7—C8—C10—N2	44.2 (4)	C11—N2—N3—C13	2.0 (3)
N1—C8—C10—N2	−161.7 (3)	C10—N2—N3—C13	178.5 (3)
N2—C11—C12—C13	2.4 (4)	C18—C17—N4—N5	−0.6 (3)
C14—C11—C12—C13	177.6 (3)	C20—C17—N4—N5	178.5 (3)
C11—C12—C13—N3	−1.3 (4)	C18—C17—N4—C16	179.4 (3)
C11—C12—C13—C15	178.6 (3)	C20—C17—N4—C16	−1.5 (5)
C7—C6—C16—O2	176.7 (3)	O2—C16—N4—N5	−170.5 (3)
C5—C6—C16—O2	−2.6 (5)	C6—C16—N4—N5	8.6 (4)
C7—C6—C16—N4	−2.3 (5)	O2—C16—N4—C17	9.5 (4)
C5—C6—C16—N4	178.3 (3)	C6—C16—N4—C17	−171.4 (3)
N4—C17—C18—C19	0.3 (3)	C18—C19—N5—N4	−0.4 (3)
C20—C17—C18—C19	−178.7 (3)	C21—C19—N5—N4	180.0 (3)
C17—C18—C19—N5	0.1 (4)	C17—N4—N5—C19	0.6 (3)
C17—C18—C19—C21	179.6 (3)	C16—N4—N5—C19	−179.4 (2)
N6—C22—C23—C24	3.7 (4)	C23—C22—N6—C26	−8.6 (4)
C30—C22—C23—C24	−170.9 (3)	C30—C22—N6—C26	166.0 (3)
C22—C23—C24—C25	2.3 (5)	C23—C22—N6—C29	−178.7 (3)
C23—C24—C25—C26	−3.5 (5)	C30—C22—N6—C29	−4.1 (4)
C24—C25—C26—N6	−1.2 (5)	C25—C26—N6—C22	7.5 (4)
C24—C25—C26—C27	177.0 (3)	C27—C26—N6—C22	−171.2 (2)
N6—C26—C27—C28	−0.6 (3)	C25—C26—N6—C29	179.5 (3)
C25—C26—C27—C28	−178.9 (3)	C27—C26—N6—C29	0.8 (3)
N6—C26—C27—C37	−176.4 (3)	C28—C29—N6—C22	170.4 (3)
C25—C26—C27—C37	5.3 (5)	C31—C29—N6—C22	−31.7 (4)
C26—C27—C28—C29	0.1 (3)	C28—C29—N6—C26	−0.8 (3)
C37—C27—C28—C29	175.2 (3)	C31—C29—N6—C26	157.1 (3)
C27—C28—C29—N6	0.4 (3)	C33—C32—N7—N8	−0.6 (4)
C27—C28—C29—C31	−156.4 (3)	C35—C32—N7—N8	179.7 (3)
C28—C29—C31—O3	128.1 (4)	C33—C32—N7—C31	178.3 (3)
N6—C29—C31—O3	−25.6 (5)	C35—C32—N7—C31	−1.5 (5)
C28—C29—C31—N7	−47.0 (4)	O3—C31—N7—N8	168.8 (3)
N6—C29—C31—N7	159.3 (3)	C29—C31—N7—N8	−15.8 (4)
N7—C32—C33—C34	0.6 (4)	O3—C31—N7—C32	−9.9 (5)
C35—C32—C33—C34	−179.7 (3)	C29—C31—N7—C32	165.4 (3)
C32—C33—C34—N8	−0.4 (4)	C33—C34—N8—N7	0.0 (4)
C32—C33—C34—C36	−178.3 (3)	C36—C34—N8—N7	178.1 (3)
C28—C27—C37—O4	−172.3 (3)	C32—N7—N8—C34	0.3 (3)
C26—C27—C37—O4	2.2 (5)	C31—N7—N8—C34	−178.6 (3)
C28—C27—C37—N9	8.3 (5)	C39—C38—N9—N10	1.2 (3)
C26—C27—C37—N9	−177.1 (3)	C41—C38—N9—N10	−179.3 (3)
N9—C38—C39—C40	−1.4 (3)	C39—C38—N9—C37	179.0 (3)
C41—C38—C39—C40	179.2 (3)	C41—C38—N9—C37	−1.5 (5)
C38—C39—C40—N10	1.2 (4)	O4—C37—N9—N10	−178.2 (3)
C38—C39—C40—C42	179.9 (3)	C27—C37—N9—N10	1.1 (4)
C2—C1—N1—C8	−179.8 (3)	O4—C37—N9—C38	4.1 (5)
C9—C1—N1—C8	3.1 (4)	C27—C37—N9—C38	−176.5 (3)
C2—C1—N1—C5	8.8 (4)	C39—C40—N10—N9	−0.4 (3)
C9—C1—N1—C5	−168.3 (3)	C42—C40—N10—N9	−179.3 (3)
C7—C8—N1—C1	−171.1 (3)	C38—N9—N10—C40	−0.5 (3)
C10—C8—N1—C1	31.0 (5)	C37—N9—N10—C40	−178.5 (3)

### Hydrogen-bond geometry (Å, º)

**Table:**

*D*—H···*A*	*D*—H	H···*A*	*D*···*A*	*D*—H···*A*
C12—H12···O2^i^	0.93	2.37	3.282 (4)	167
C21—H21*B*···O1^ii^	0.96	2.59	3.404 (4)	142
C30—H30*C*···N3^iii^	0.96	2.52	3.472 (4)	169
C33—H33···O4^iv^	0.93	2.55	3.393 (4)	152

Symmetry codes: (i) *x*, *y*−1, *z*; (ii) −*x*+3/2, *y*, *z*−1/2; (iii) *x*−1/2, −*y*+1, *z*; (iv) *x*, *y*+1, *z*.
